# Sequencing and Analysis of the Entire Genome of the Mycoparasitic Bioeffector Fungus Trichoderma asperelloides Strain T 203 (Hypocreales)

**DOI:** 10.1128/mra.00995-21

**Published:** 2022-02-17

**Authors:** Maggie Gortikov, Zheng Wang, Andrei S. Steindorff, Igor V. Grigoriev, Irina S. Druzhinina, Jeffrey P. Townsend, Oded Yarden

**Affiliations:** a Department of Plant Pathology and Microbiology, The Robert H. Smith Faculty of Agriculture, Food and Environment, The Hebrew University of Jerusalem, Rehovot, Israel; b Department of Biostatistics, Yale University, New Haven, Connecticut, USA; c U.S. Department of Energy Joint Genome Institute, Lawrence Berkeley National Laboratory, Berkeley, California, USA; d Department of Plant and Microbial Biology, University of California, Berkeley, Berkeley, California, USA; e Fungal Genomics Laboratory (FungiG), Nanjing Agricultural University, Nanjing, China; f Department of Ecology and Evolutionary Biology, Yale University, New Haven, Connecticut, USA; University of California, Riverside

## Abstract

The filamentous mycoparasitic fungus Trichoderma asperelloides (Hypocreales, Ascomycota, Dikarya) strain T 203 was isolated from soil in Israel by the Ilan Chet group in the 1980s. As it has been the subject of laboratory, greenhouse, and field experiments and has been incorporated into commercial agricultural preparations, its genome has been sequenced and analyzed.

## ANNOUNCEMENT

The mycoparasitic strain Trichoderma asperelloides T 203 (=TH 203), formerly identified as Trichoderma asperellum and prior to that Trichoderma harzianum, is a cryptic sister species to *T. asperellum* (1). Its fungal host range, host recognition traits, and mechanistic aspects of the mycoparasite interactions have been extensively studied (2–4). The strain has also been shown to confer transient repression of the plant immune response, followed by enhanced root colonization and eventual stimulation of plant growth and resistance to a wide range of adverse environmental conditions, including salt stress (5–8). The potential to use this strain as a biocontrol agent has been repeatedly examined under greenhouse and field conditions (9).

**FIG 1:**
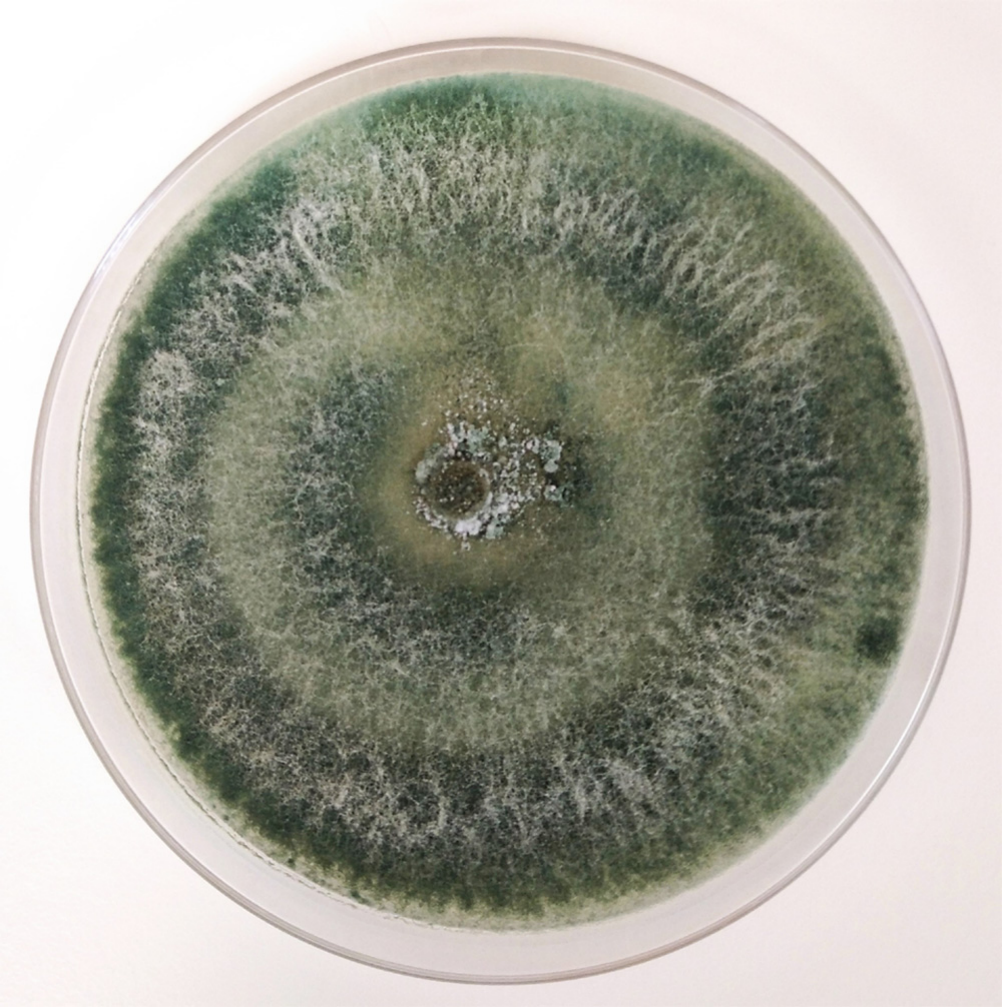
*Trichoderma asperelloides* strain T 203 cultured for 1 week on potato dextrose agar (PDA) at 28°C.

DNA was extracted from a freeze-dried, powdered culture of *T. asperelloides* T 203 (Fig. 1) grown in potato dextrose broth for 1 week, using a modified cetyltrimethylammonium bromide (CTAB)-based method, followed by chlorophorm:octanol extraction and isopropanol precipitation steps (10–12). The DNA yield and quality were assessed using the Synergy HTX multimode reader (Biotek, VT, USA) and verified by DNA electrophoresis. The sequencing library was built using the IDT Lotus DNA library prep kit (part number 10001074), full-length dual barcode adapters were ligated, and the quality of the fragments was checked using the high-sensitivity D5000 tapes (part number 5067-5592) on the Agilent TapeStation 4200 system and the KAPA library quantification kit (catalog number 07960298001), respectively. Genome sequencing was carried out on the Illumina NovaSeq S4 platform with 2 × 151-bp reads. A total of 52 million raw reads were produced for the *T. asperelloides* samples. The raw reads (read length, 2 × 150 bp) were processed using Trim Galore v0.6.6 (https://github.com/FelixKrueger/TrimGalore). An enriched set of mitochondrial reads was then extracted from the original input fastq reads by kmer matching using BBDuk in BBTools v38.44, using defaults, against the *Trichoderma* organelle contigs available at GenBank/NCBI. The matching reads were used to assemble the mitochondrial genome using SPAdes v3.15.2 (13). A similar methodology employing the UNITE ribosomal DNA (rDNA) database (14) was used to reassemble the rDNA from the filtered reads. Finally, an assembly of the target genome was generated using the resulting nonmitochondrial reads with SPAdes (13) using the following parameters: --phred-offset 33 --cov-cutoff auto -t 12 -m 32 --careful. The assembly size was 36,270,279 bp, with 100× coverage. The assembly comprised 354 genomic scaffolds; the *L*_50_ value was 0.29 Mbp, the *N*_50_ value was 35 bp, and the GC content was 47.98%. The genome assembly was annotated using the JGI Annotation Pipeline (15), which combines several gene predictions and annotation methods with transcriptomics data and integrates the annotated genomes into MycoCosm (https://mycocosm.jgi.doe.gov), a Web-based fungal resource for comparative analysis (15). The completeness of the genome annotation was assessed using BUSCO v4.0.6 (16) using the hypocreales_odb10 database, resulting in 97.5% (single copy, 97.2%; duplicate, 0.3%) completeness.

The genome of *T. asperelloides* T 203 will contribute to the understanding of genome evolution within the genus *Trichoderma* as well as to the understanding of the comparative genome organization and diversity among strains of *T. asperelloides*, a species under continuous study for both fundamental science as well as commercial domestication.

### Data availability.

This whole-genome shotgun sequence of *T. asperelloides* T 203 has been deposited at DDBJ/ENA/GenBank under BioProject accession number PRJNA772304 with BioSample accession number JAJKFY000000000. The raw reads can be found under SRA accession number SRR17157108.
